# Field Evaluation of Polymer Capacitive Humidity Sensors for Bowen Ratio Energy Balance Flux Measurements

**DOI:** 10.3390/s100807748

**Published:** 2010-08-20

**Authors:** Michael J. Savage

**Affiliations:** Soil-Plant-Atmosphere Continuum Research Unit, School of Environmental Sciences, University of KwaZulu-Natal, Pietermaritzburg, 3201, South Africa; E-Mail: savage@ukzn.ac.za; Tel.: +27-33-260-5514

**Keywords:** humidity resolution, energy balance fluxes, humidity measurement, surface-layer scintillometer, eddy covariance

## Abstract

The possibility of reliable, reasonably accurate and relatively inexpensive estimates of sensible heat and latent energy fluxes was investigated using a commercial combination thin-film polymer capacitive relative humidity and adjacent temperature sensor instrument. Long-term and unattended water vapour pressure profile difference measurements using low-power combination instruments were compared with those from a cooled dewpoint mirror hygrometer, the latter often used with Bowen ratio energy balance (BREB) systems. An error analysis, based on instrument relative humidity and temperature errors, was applied for various capacitive humidity instrument models. The main disadvantage of a combination capacitive humidity instrument is that two measurements, relative humidity and temperature, are required for estimation of water vapour pressure as opposed to one for a dewpoint hygrometer. In a laboratory experiment using an automated procedure, water vapour pressure differences generated using a reference dewpoint generator were measured using a commercial model (Dew-10) dewpoint hygrometer and a combination capacitive humidity instrument. The laboratory measurement comparisons showed that, potentially, an inexpensive model combination capacitive humidity instrument (CS500 or HMP50), or for improved results a slightly more expensive model (HMP35C or HMP45C), could substitute for the more expensive dewpoint hygrometer. In a field study, in a mesic grassland, the water vapour pressure measurement noise for the combination capacitive humidity instruments was greater than that for the dewpoint hygrometer. The average water vapour pressure profile difference measured using a HMP45C was highly correlated with that from a dewpoint hygrometer with a slope less than unity. Water vapour pressure measurements using the capacitive humidity instruments were not as accurate, compared to those obtained using a dewpoint hygrometer, but the resolution magnitudes for the profile difference measurements were less than the minimum of 0.01 kPa required for BREB measurements when averaged over 20 min. Furthermore, the longer-term capacitive humidity measurements are more reliable and not dependent on a sensor bias adjustment as is the case for the dewpoint hygrometer. A field comparison of CS500 and HMP45C profile water vapour pressure differences yielded a slope of close to unity. However, the CS500 exhibited more variable water vapour pressure measurements mainly due to its increased variation in temperature measurements compared to the HMP45C. Comparisons between 20-min BREB sensible heat fluxes obtained using a HMP45C and a dewpoint hygrometer yielded a slope of almost unity. BREB sensible heat fluxes measured using a HMP45C were reasonably well correlated with those obtained using a surface-layer scintillometer and eddy covariance (slope of 0.9629 and 0.9198 respectively). This reasonable agreement showed that a combination capacitive humidity instrument, with similar relative humidity (*RH*) and temperature error magnitudes of at most 2% *RH* and 0.3 °C respectively, and similar measurement time response, would be an adequate and less expensive substitute for a dewpoint hygrometer. Furthermore, a combination capacitive humidity instrument requires no servicing compared to a dewpoint hygrometer which requires a bias adjustment and mirror cleaning each week. These findings make unattended BREB measurements of sensible heat flux and evaporation cheaper and more reliable with the system easier to assemble and service and with reduced instrument power.

## 1. Introduction

The use of the Bowen ratio energy balance (BREB) method for measurement of sensible heat and latent energy fluxes has a long historical record. Theoretical aspects of the BREB method in relation to data exclusion have been examined [1–3]. The method has been used for specific purposes to estimate evaporation for different canopy surfaces [4–18] including grassland, mulch-covered bare soil [19] and open water [20,21]. The method applied requires accurate measurement of air temperature and water vapour pressure differences between two vertical (profile) positions above the surface of interest.

Cellier and Olioso [22] concluded that a BREB system using a single combination capacitive humidity and temperature instrument for measurements of water vapour pressure for both heights yields good flux estimates with an instrument power consumption of 1 to 10 mA compared to 100 to 200 mA for a dewpoint hygrometer system and 100 to 500 mA for a ventilated wet- and dry-bulb psychrometer system. They compared BREB fluxes with those obtained using a one-dimensional sonic anemometer eddy covariance (EC) system.

While the EC method is the *de facto* standard for flux measurements, instrument cost and a greater power requirement may prohibit simultaneous measurements within the same or in different catchments over different canopy types or for simultaneous flux measurements above areas subjected to different management. Furthermore, EC measurements require many corrections [23] and there have also been numerous reports of flux underestimation and lack of energy balance closure [24] with different methods proposed for adjusting the measured EC fluxes, some of which depend on the Bowen ratio [25]. The amount of data collected, and the required expertise, is not as demanding for the BREB method compared to that for EC. Nevertheless, EC is regarded as the reference method for turbulent heat flux measurements.

BREB automatic height exchange [14] and fixed-level systems that use two combination capacitive humidity and temperature instruments have been described [19]. BREB systems using a single combination capacitive humidity and temperature instrument shared between two heights have also been used [14,22]. Recent improvements in the accuracy of polymer capacitive relative humidity sensors from within 3% relative humidity (RH) to within 1.5% RH encourage further investigation of such instruments for BREB flux measurements. Furthermore, a full error analysis of the use of a single combination capacitive humidity and temperature instrument in a BREB system has not been reported on and BREB flux comparisons for such systems against other flux methods have been rare and only for limited duration.

Based on previous experiences and personal communications with other researchers, the dewpoint hygrometer of the BREB system used for long-term field measurements may after some time yield unreliable data due to the sensor's inability to maintain a stable bias. Given other practical problems such as servicing and power requirements for dewpoint hygrometer systems employed in distant locations, the objective of this work was to investigate the use of a BREB system, by substituting the dewpoint hygrometer with a combination thin-film polymer capacitive relative humidity and air temperature instrument, for long-term and unattended estimation of sensible heat and latent energy fluxes. An error analysis is presented for two different combination capacitive humidity instrument models, which are compared to a dewpoint hygrometer, in the laboratory and in a mesic grassland, for their ability to measure water vapour pressure profile differences for an extended period. Furthermore, to test the adequacy of the combination capacitive humidity instruments used in the field for flux measurements, comparisons of BREB-measured sensible heat flux against that obtained using the path-weighting surface-layer scintillometer (SLS) method, dependent on Monin-Obukhov Similarity Theory (MOST), and the standard EC method are presented. Such comparisons have not been presented previously in the literature.

## 2. Theoretical Considerations

### 2.1. BREB Theory

A brief description of the BREB method for measuring sensible and latent energy flux is presented. The BREB method relies on the K-theory hypothesis for which sensible heat flux, for example, is proportional to an exchange coefficient for sensible heat flux and the profile air temperature gradient.

Bowen [26], building on previous work of Cummings [27], John Dalton and others [28], attempted to obtain expressions for latent energy flux in terms of water vapour pressure gradients above a surface. The latent energy flux *LE* (W m^−2^) is given by:

(1) $$\mathrm{LE}=\left({\rho c}_{p}/\gamma \right)K_{w}\left(\partial \bar{e}/\partial z\right)=\left({\rho c}_{p}/\gamma \right)K_{w}\left(\bar{e_{2}}-\bar{e_{1}}\right)/\left(z_{2}-z_{1}\right)$$

where *ρ* is the density of air (kg m^−3^), *c_p_* the specific heat capacity of air at constant pressure (J kg^−1^ K^−1^), *γ* the psychrometric constant (approximately 0.066 kPa K^−1^ at sea level), *K_w_* the exchange coefficient (m^2^ s^−1^) for latent energy flux, and $\bar{e_{2}}$ and $\bar{e_{1}}$ the time-averaged water vapour pressures (kPa) at heights *z*_2_ and *z*_1_ above the soil surface respectively where $\left(\bar{e_{2}}-\bar{e_{1}}\right)/\left(z_{2}-z_{1}\right)$ is the water vapour pressure profile gradient. The averaging period is usually 20 min, 30 min or hourly although even 5 min periods have been used [29].

Similarly, the sensible heat flux *H* is given by:

(2) $$H={\rho c}_{p}K_{h}\left(\partial \bar{T}/\partial z\right)={\rho c}_{p}K_{h}\left(\bar{T_{2}}-\bar{T_{1}}\right)/\left(z_{2}-z_{1}\right)$$

where *K_h_* is the exchange coefficient for sensible heat flux (m^2^ s^−1^) and $\bar{T_{2}}$ and $\bar{T_{1}}$ the time-averaged air temperatures (°C) at heights *z*_2_ and *z*_1_ above the soil surface respectively where $\left(\bar{T_{2}}-\bar{T_{1}}\right)/\left(z_{2}-z_{1}\right)$ is the air temperature profile gradient. The sign convention used is that under normal conditions during daytime hours, canopy- to atmosphere-directed *H* and *LE* fluxes are indicative of energy losses at the surface and are negative.

Following Bowen [26], the ratio *β* is defined as:

(3) β=H/LE

and using Equations 1 and 2, it can be written as:

(4) $$\beta =\left(\gamma K_{h}/K_{w}\right)\left(\bar{T_{2}}-\bar{T_{1}}\right)/\left(\bar{e_{2}}-\bar{e_{1}}\right).$$

hence *β* can be determined by measuring air temperature and water vapour pressure at two levels in the atmosphere. Ignoring photosynthesis, advection and physically and biochemically stored fluxes, the surface energy balance is given by:

(5) $$R_{n}=-\mathrm{LE}-H-S$$

where *R_n_* is the net irradiance and *S* the soil heat flux. Combining Equations 3 and 5:

(6) $$\mathrm{LE}=-\left(R_{n}+S\right)/\left(1+\beta \right)$$

(7) $$H=-\beta \left(R_{n}+S\right)/\left(1+\beta \right)$$

where:

(8) $$\beta =\gamma \left(\bar{T_{2}}-\bar{T_{1}}\right)/\left(\bar{e_{2}}-\bar{e_{1}}\right)$$

if it is assumed in Equation 4, as is commonly the case [30], that *K_w_* = *K_h_*. The equality of these two exchange coefficients, referred to as the Similarity Principle, allows the BREB method to be used without the need for atmospheric stability adjustments. A limitation of the BREB method occurs when the denominator of Equations 6 and 7 approaches zero. Data exclusion details associated with this situation are discussed by Savage *et al*. [3].

The BREB system, which includes field measurements of *R_n_* and *S*, is used mainly for determining *H* and *LE*. BREB systems are critically dependent on the profile water vapour pressure and air temperature difference measurements. Limitations of use of a cooled dewpoint mirror hygrometer in BREB systems for measurement of water vapour pressure include an increased power requirement and the requirement that the electronic bias of the sensor is adjusted regularly—usually weekly.

### 2.2. Resolution of BREB Sensors Used

In addition to other measurements, the BREB method ideally requires, for example, a dewpoint hygrometer or combination humidity and temperature instrument yielding a water vapour pressure resolution magnitude less than 0.01 kPa. The stability of the dewpoint measurements for the commercially available Dew-10 hygrometer used in this study (Table 1) is reportedly within 0.05 °C, resulting in a water vapour pressure resolution magnitude less than 0.01 kPa for temperatures less than 26 °C.

Between 26 and 40 °C, Dew-10 dewpoint measurements within 0.05 °C result in a water vapour pressure resolution magnitude less than 0.02 kPa. Currently, combination humidity and temperature instruments do not yield a water vapour pressure resolution magnitude of less than 0.01 kPa. This investigation however, examines whether water vapour pressure difference measurements within 0.01 kPa are possible. The limitation in the measurement of the profile difference in air temperature is the datalogger temperature resolution magnitude of 0.006 °C in this study. This is normally achievable using a pair of unshielded 75 μm type-E thermocouples or shielded and interchanging aspirated temperature sensors.

### 2.3. Error Analysis of Polymer Capacitive Humidity Instruments

The error in water vapour pressure for a dewpoint hygrometer is dependent on the random error, bias and resolution of the measured dewpoint. The one disadvantage of using a combination polymer capacitive relative humidity and air temperature instrument, compared to a dewpoint hygrometer, is that the calculated water vapour pressure depends on relative humidity and temperature measurements from two spatially-separated sensors, each mounted on different chips, with both measurements having their own random error, bias and resolution limitations. One commercial model combination capacitive humidity instrument however has both sensors attached to the same chip, thereby allowing both measurements at virtually the same position of the airstream. Ideally, for spatially-separated relative humidity and temperature sensors when calculating water vapour pressure from the measured relative humidity, the temperature of the humidity sensor needs to be known and not that of the temperature sensor. Usually however, the two sensors are located in very close proximity—millimetres apart. The humidity chamber can be thermally insulated but this alone may not eliminate all differences in temperature between the capacitive humidity and temperature sensor. The temperature difference between the relative humidity and temperature sensor should decrease with increase in flow rate in the chamber but this would increase the system current drain.

The HMP45C (Table 1) combination polymer capacitive humidity and temperature instrument contains a Vaisala Humicap 180 capacitive humidity sensor (Vaisala, Helsinki, Finland) and a platinum resistance thermometer. The relative humidity sensor accuracy is within 2% relative humidity (*RH*) where the % unit is in *RH* units and not a percentage of the measured *RH* (for *RH* between 0 and 90%) and temperature accuracy is within 0.2 °C to 20 °C (Table 1). For the Campbell CS500 combination instrument, which contains a Vaisala Intercap capacitive relative humidity sensor and a platinum resistance thermometer, the accuracy in *RH* measurements is within 3% *RH* and temperature accuracy is within 0.46 °C at 20 °C. The replacement for the CS500, the Campbell HMP50, has almost identical sensor specifications. The limitation of water vapour pressure calculated is the accuracy and the resolution of the measurements for the relative humidity and temperature sensors. Based on manufacturer information, the magnitude of the error in temperature, |*δT*| (°C), for the HMP45C is given by:

for *T* ≥ 20 °C
  (9) |δT|=0.2+0.1(T−20)/20
and for *T* ≤ 20 °C
  (10) |δT|=0.2+0.1(20−T)/20
and that for a CS500 for *T*≥ 0 °C is given by:
  (11) |δT|=0.3+0.008T.

**Figure 1.**
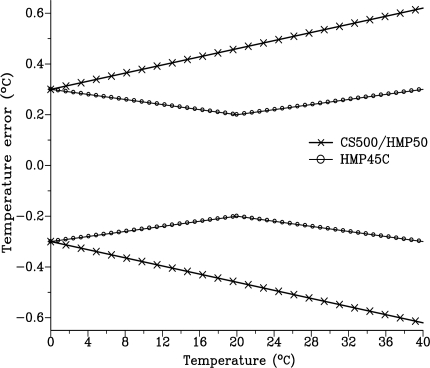
The error in temperature (°C) for the two combination polymer capacitive relative humidity and temperature instruments. The error for any given temperature is between the upper and lower corresponding set of sensor curves.

These relationships are depicted in Figure 1. Between 0 and 40 °C, |*δT*| for the CS500 varies linearly from 0.3 to 0.62 °C respectively compared to 0.3 °C for the HMP45C for the same temperature range but with a minimum for |*δT*| of 0.2 °C at 20 °C. Therefore, the HMP45C has a more accurate temperature sensor than that used in the CS500.

The water vapour pressure of an airstream, *e* (kPa), is calculated from the measured capacitive sensor *RH* and the saturation water vapour pressure *e_s_* (kPa) calculated from the adjacent temperature sensor using:

(12) $$e=\mathrm{RH}\times e_{s}/100$$

where:

(13) $$e_{s}=0.6108\;\text{exp}\left(17.2694\;T/\left(237.3+T\right)\right)$$

and where *T* (°C) is the sensor-measured airstream temperature. Hence the error magnitude in water vapour pressure, *δe* (kPa), is estimated from the error in *RH*, *δRH*, and error in *e_s_*, *δe_s_* (kPa), using:

(14) $$\delta e=\sqrt{{\left(\partial e/\partial \mathrm{RH}\right)}^{2}{\delta \mathrm{RH}}^{2}+{\left(\partial e/{\partial e}_{s}\right)}^{2}{\delta e}_{s}^{2}}$$

where *∂e*/*∂RH* = *e_s_*/100 and *∂e*/*∂e_s_* = *RH*/100 and where *∂e_s_* = (*∂e_s_*/∂*T*)·*δT* = Δ·*δT* where Δ (kPa °C^−1^) is the slope of the saturation water vapour pressure *vs* temperature relationship at temperature *T*:

(15) $$\Delta =4098.02862\;e_{s}/{\left(237.3+T\right)}^{2}.$$

Finally then, from Equation 14, the error in water vapour pressure *δe* for any relative humidity and temperature combination sensor is given by:

(16) $$\delta e=\sqrt{{\left(e_{s}/100\right)}^{2}{\delta \mathrm{RH}}^{2}+{\left(\mathrm{RH}/100\right)}^{2}\Delta^{2}\delta T^{2}.}$$

In Equation 16, due mainly to the role of Δ, the first term on the right hand side within the square root, dictated by *e_s_* and *δRH*, is usually much greater than the second term except when temperature is low and *RH* is high. Thus the error *δe*, mainly governed by *δRH*, increases with temperature. The error *δe* is temperature dependent since both *e_s_* and Δ are temperature dependent and the information provided by the manufacturer shows that *δT* is also temperature dependent. For the HMP45C, combining Equations 9, 10 and 16:

(17) $${\delta e}_{\text{HMP45C}}=\sqrt{{\left(e_{s}/100\right)}^{2}{\delta \mathrm{RH}}^{2}+{\left(\mathrm{RH}/100\right)}^{2}\Delta^{2}{\left(0.2+0.1\;(T-20)/20\right)}^{2}}$$

for *T* ≥ 20 ^°^C or for *T* < 20 ^°^C

(18) $${\delta e}_{\text{HMP45C}}=\sqrt{{\left(e_{s}/100\right)}^{2}{\delta \mathrm{RH}}^{2}+{\left(\mathrm{RH}/100\right)}^{2}\Delta^{2}{\left(0.2+0.1\;(20-T)/20\right)}^{2}}$$

and for the CS500, combining Equations 11 and 16:

(19) $$\delta e_{\text{CS500}}=\sqrt{{\left(e_{s}/100\right)}^{2}{\delta \mathrm{RH}}^{2}+{\left(\mathrm{RH}/100\right)}^{2}\Delta^{2}{\left(0.3+0.008\;T\right)}^{2}}$$

for *T* ≥ 0 °C.

**Figure 2.**
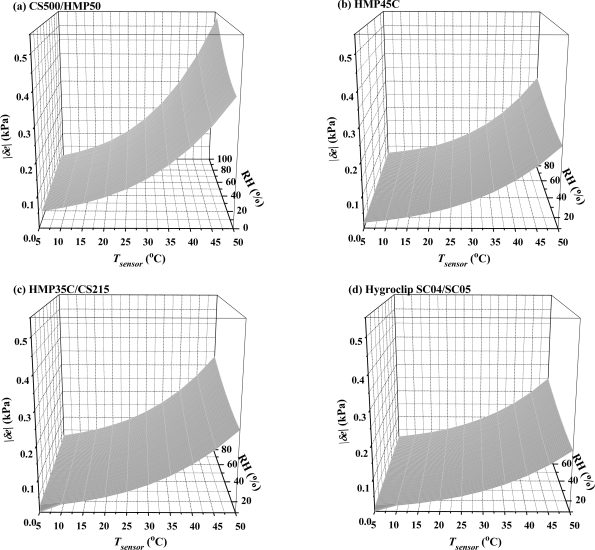
The magnitude of the error in the water vapour pressure |*δe*| for various polymer capacitive humidity combination instruments as a function of relative humidity and air temperature based on the manufacturer's specification: (a) CS500; (b) HMP45C; (c) HMP35C/CS215; (d) Hygroclip SC04/SC05.

Using the manufacturer's specification for the HMP45C that *δRH* = ±2% (for 0% ≤ RH ≤ 90%) and for the CS500 *δRH* = ±3% (for 10% ≥ *RH* ≥ 90%), |*δe*| was determined as a function of temperature between 5 and 50 °C and *RH* between 10 and 90 %. The calculations show (Figures 2(a,b)) that |*δe*| far exceeds the 0.01 kPa limit required by the BREB method and attainable by the dewpoint hygrometer. However, these error magnitudes should be regarded as the maximum error in water vapour pressure. For temperatures between 15 and 40 °C, |*δe*_HMP45C_| ranges from 0.03 kPa to 0.15 kPa (for a *RH* of 50 %, Figure 2(b)). The error magnitudes for the CS500 are 50 % greater, and more so for high relative humidities and temperatures exceeding 30 °C (Figure 2(a)).

One option for reducing the error magnitudes shown in Figure 2 is to reduce enclosure temperature to less than 30 °C. While it may be possible to reduce enclosure temperature, which contributes to the hygrometer chamber temperature, it is not possible to reduce the temperature of the air flowing through the chamber.

If the same combination capacitive humidity and temperature instrument is used for determining water vapour pressure measurements for both BREB heights, and if it can be assumed that the error magnitudes shown in Figure 2 and the systematic bias in sensor data are almost the same for both heights, then the error in water vapour pressure would tend to cancel out. However, laboratory measurements would be required to prove this before the instrument could be used in the field for BREB measurements. Furthermore, if the airstream temperature of the two levels is different, as might be expected for large sensible heat fluxes directed away from the surface, the error in *e* at both levels may not cancel due to the non-linear nature of *δe* as a function of temperature (Equation 16).

The error for other combination humidity and temperature instruments, based on manufacturer specifications, which were not used for measurements in this study, are as follows: Hygroclip SC04/SC05 (Table 1) *δRH* = ±1.5% in *RH* % units and *δT* = ±0.3 °C; Campbell CS215 (a low-power digital relative humidity and temperature sensor with SDI-12 capability): *δRH* = ±2 % and *δT* = ±0.4 °C (according to Campbell although Sensirion state *δRH* = ±1.8 % and *δT* = ±0.3 °C). The *RH* and *T* errors for the CS215 are similar to those for the Campbell HMP35C combination instrument (which contains a Vaisala capacitive polymer H relative humidity sensor and a thermistor) except that the CS215 contains a single-chip element that includes both relative humidity and temperature sensors. The Campbell 155A combination instrument is based on a Vaisala model 180 Humicap capacitive humidity sensor and a platinum resistance thermometer. Measurement errors are specified in Table 1, for analogue measurements. Based on these error values, |*δe*| values for the CS215 are marginally greater than those for the HMP45C (Figure 2(b),(c)) and |*δe*| for the Hygroclip and the HMP155A instruments are marginally reduced (Figure 2(d)) compared to the HMP45C. The error magnitudes for the CS500/HMP50 combination instrument are the greatest of those considered (Figure 2(a)). An aspect not considered in the error analysis is the water vapour pressure resolution.

## 3. Materials and Methods

### 3.1. Laboratory Water Vapour Pressure Difference Measurements

In the laboratory, a LI610 reference dewpoint generator (Table 1), controlled by a Campbell 21X datalogger, was used. The datalogger controlled the dewpoint generator to increment the dewpoint temperature generated in an airstream by 0.1 °C every 2 min. The datalogger resolution for the dewpoint hygrometer water vapour pressure measurements was 0.0001 kPa. Dewpoints ranged between 0 °C and a degree below room temperature, the latter typically 21 °C, after which the datalogger reset the generator dewpoint to 0 °C. In this way, unattended reference water vapour pressure differences were compared against those measured using the various instrument types. Two new, factory calibrated, combination capacitive humidity and air temperature instruments, a HMP35C and a CS500, and a new Dew-10 dewpoint hygrometer, were used with the same methodology applied for each of the three measurement comparison runs except that for the Dew-10, an additional pump was used at the outlet to pump air from the dewpoint generator to the mirror and out to the atmosphere. For each run, a capacitive humidity or dewpoint instrument was placed in a 50-mm diameter and 300-mm long cylindrical rigid-plastic tube, open at one end. The open end was filled with non-water retaining sponge material with the other end connected, using a short length of hose that was thermally insulated, to the dewpoint generator. For each calibration run, the relevant measurements were sampled every 1 s and averages obtained for the last 80 s of a 2-min period. The average water vapour pressure difference between two consecutive 2-min periods were calculated and averaged every 20-min.

### 3.2. Climate of the Field Site

Field measurements were conducted in a mesic grassland in a summer rainfall area at the Hay Paddock site neighbouring Ashburton and close to the suburb of Bellevue of Pietermaritzburg, South Africa (29°38′S, 30°26′E) with an altitude of 671.3 m. The long-term annual rainfall for Pietermaritzburg is 928 mm (58 years of data) and the mean maximum and mean minimum air temperatures are 24.8 and 12.3 °C respectively (South African Weather Service). During winter, there was frequent dewfall and occasional frost. Management practices at the research site included mowing (normally in April each year) and burning (in August when firebreaks are established and in October).

## 3.3. BREB Field Measurements

BREB field measurements were conducted from January to July 2004. Water vapour pressure profile difference comparisons were made using a Dew-10 dewpoint hygrometer and two combination capacitive humidity instruments, the HMP45C and CS500. The minimum fetch distance for the site for the prevailing wind direction was 135 m for the BREB systems. The mixed grassland area to the south of the site was more exposed with a slight slope increase. The adjacent area had occasional trees. To the north of the study site, there was a residential area and occasional tall trees.

BREB systems, modified from Campbell 023A systems [31] and connected to a 21X datalogger, were used to measure air temperature and water vapour pressure profile differences between heights of 1.55 and 2.96 m above the soil surface using HMP45C and CS500 combination instruments and a Dew-10 hygrometer. The 023A system consisted of two 2-L mixing bottles, one for air from each measurement height, with a time constant of 5 min for a flow rate of 0.4 L min^−1^. Air continuously sampled from each height was drawn to the respective mixing bottle housed in the instrument enclosure and then to the chamber containing either the Dew-10 or combination humidity instrument.

Each capacitive humidity instrument was sealed in a metal chamber. Unlike the dewpoint hygrometer chamber, the capacitive humidity chambers were heavily insulated using layers of mirror tape to dampen sudden changes in temperature. Tubing in the vicinity of the chamber was covered with adhesive aluminium tape in an attempt to further reduce temperature differences between the humidity and temperature sensors and that of the airstream. The Bev-a-line tubing (Cole-Parmer, Vernon Hills, IL, USA) used for the water vapour pressure measurements was as short as possible to minimise the transit time of the air from the intake point to the instrument. Furthermore, the filter cover of the combination capacitive humidity instruments was removed to improve the time response to relative humidity. The inlet Teflon filter (1-μm pore size) at each of the two intake positions was however retained for both BREB capacitive humidity systems to prevent entry of liquid water and dirt. The voltages corresponding to the HMP45C relative humidity and air temperature were differential. The corresponding CS500 voltages were single-ended, as recommended by the manufacturer. Every 1 s, the airstream atmospheric water vapour pressure was calculated from the measured relative humidity and temperature. Averages and standard deviations were calculated every 20 min.

In the first field experiment, the HMP45C and CS500 instruments were placed in individual humidity chambers. The outlet of the one chamber was connected to the inlet of the other. The inlet of the first chamber was connected to the inlet airstream from the mixing bottles and the outlet of the second bottle was connected to the inlet of the pump. The outlet of the pump was vented to the atmosphere. In this fashion, the two instruments were exposed to the same water vapour pressure.

In a second experiment, measurements were made using two chambers connected in-line with the one chamber containing a HMP45C and the second chamber containing a Dew-10 dewpoint hygrometer. Most of the measurements were with the inlet air passing through the HMP45C chamber first and then entering the dewpoint hygrometer chamber and then out to the pump. This arrangement would reduce the influence of the dewpoint hygrometer measurement method on the HMP45C measurements. The dewpoint hygrometer required a weekly bias adjustment for convergence on the correct dewpoint. The capacitive humidity instruments required no attention.

The air temperature differences between two levels were measured using a pair of naturally-ventilated and unshielded 75-μm type-E thermocouples. At each level, a parallel combination of 75-μm thermocouples was used. Extra insulation was used to cover the thermocouple connectors at the thermocouple join. Extra precautions were taken by covering and thermally insulating the point at which the thermocouple wires were connected to the datalogger. The thermocouples were inspected for damage, cleanliness, insects and cobwebs during each site visit and replaced or cleaned.

For measuring the remaining components of the energy balance, required for calculating *H* and *LE* (Equations 6 and 7 respectively), three Q*7 [Radiation and Energy Balance System (REBS), Seattle, WA, USA] net radiometers placed at 2 m above the soil surface were used to measure net irradiance. Seven soil heat flux plates (model HFT-3, REBS) were used to measure soil heat flux at a depth of 80 mm. A system of parallel thermocouples at depths of 20 and 60 mm were used to measure soil temperature. Volumetric soil water content in the first 60 mm from the soil surface was measured using a frequency domain reflectometer (ThetaProbe, model ML2x, Delta-T Devices, Cambridge, UK) and a Campbell 615 soil reflectometer. These soil measurements were required to calculate the soil heat flux stored above the plates [32]. A measurement scan rate of 1 s was used and averages obtained every 2 min which were in turn used to calculate 20-min averages for the BREB calculations. The net radiometers and soil heat flux plates and the EC system were positioned approximately midway between the transmitter and receiver units of the SLS.

### 3.4. Surface-Layer Scintillometer

The methodology and details for the dual-beam SLS measurements (model SLS40-A, Scintec Atmospärenmessetechnik, Tübingen, Germany) for calculating sensible heat flux (*H*) have been presented [33–36] and are only briefly described here. The SLS employs a diode laser source with an output wavelength of 670 nm and 1 mW (2 mW peak) mean output power. The SLS40-A receiver has four detectors, two of which are used for automatic identification of and correction for transmitter vibration by the software used for analysis [35]. The detectors also allow for correction of beam intensity caused by beam movement. The SLS system allows on-line beam transmission measurements at a frequency of 1 kHz and subsequent calculation of *H* every 20 min by application of the semi-empirical MOST. A beam distance of 101 m was used. The SLS was positioned at a height of 1.68 metres above the soil surface. The SLS signal processing unit was connected to a computer via the computer serial port. Vegetation height, weighted over the beam path length [34], varied seasonally but was always less than 1.13 m.

### 3.5. Eddy Covariance

A three-dimensional sonic anemometer (SWS-211/3V, Applied Technologies, Boulder, CO, USA) was used as an EC system to measure *H* at a height of 2.12 m above the soil surface. This anemometer, with a 100-mm sonic path length, was connected to a digital to analogue converter that then connected to a Campbell 21X datalogger. Measurements of the three components of wind velocity, *u*, *v*, *w* in the *x*, *y* and *z* directions respectively, and sonic temperature *T_sonic_* were performed every 0.1 s (frequency of 10 Hz). The sonic anemometer measurements were processed on-line. No coordinate rotations [23] were possible, since no storage equipment for the high frequency data was available. The covariance between *w* and *T_sonic_*, for determining *H*, was calculated using $H=-\rho c_{p}\bar{w^{\prime }{T^{\prime }}_{\mathrm{sonic}}}$ where the primes indicate fluctuations from the respective 20-min means.

## 4. Results and Discussion

Before the results of the flux measurements for the various field methods could be compared, a study was undertaken to investigate the possible substitution of the dewpoint hygrometer by a combination capacitive humidity instrument.

### 4.1. Laboratory Use of Combination Polymer Capacitive Humidity Instruments for Water Vapour Pressure Profile Difference Measurement

**Figure 3.**
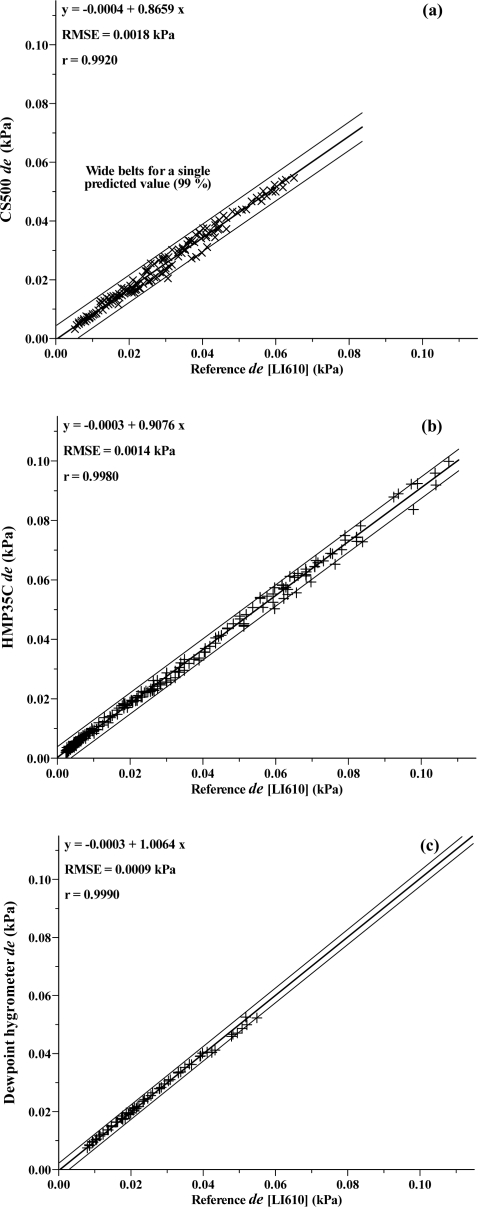
Regression plots of the reference water vapour pressure differences in kPa (*de*) measured using the LI610 Dewpoint Generator, compared with the measured water vapour differences (*de*) using: (a) CS500; (b) HMP35C; (c) Dew-10.

In the laboratory, profile differences in water vapour pressure (*de*) were simulated using a LI610 dewpoint generator. The *de* values generated were compared against measurements in three separate experiments using a Dew-10 dewpoint hygrometer and HMP35C and CS500 combination capacitive humidity instruments. Judging by the root mean square error (RMSE) for the three measurement comparisons (Figure 3), *de* measurements using the CS500 (Figure 3a) exhibited greatest variability (Figure 3(b),(c)).

The ability of the HMP35C to measure very small *de* values appears good in this laboratory experiment in which airstream temperature had a much reduced diurnal range compared to that experienced in the field. The slope is close to 1 (Figure 3(b)) but somewhat less than that for the Dew-10 dewpoint hygrometer *vs* reference *de* comparison (Figure 3(c)). In the case of the Dew-10 measurements, the normal BREB pump was also used to pump air from the dewpoint calibrator across the mirror and out to the atmosphere. In the case of the CS500 and HMP35C measurements, the BREB pump was not used. The slope of less than 1 for these units, compared to close to 1 for the Dew-10 measurements indicates the need for the additional BREB pump. The Dew-10 *vs* reference *de* comparison had the smallest RMSE (Figure 3c) but the Dew-10 is more expensive and suffers from the problems of long-term maintenance of a stable bias as mentioned previously. The HMP35C, now replaced by the HMP45C with almost identical sensor specifications, seemed a promising substitute for the dewpoint hygrometer.

Of particular note is that in spite of the error in water vapour pressure for combination capacitive humidity instruments, the resultant *de* resolution is within 0.005 kPa or better (Figure 3(a),(b)), when compared with the dewpoint generator measurements, under fairly temperature-controlled conditions.

### 4.2. Field Use of Combination Polymer Capacitive Humidity Instruments for Measurement of de

**Figure 4.**
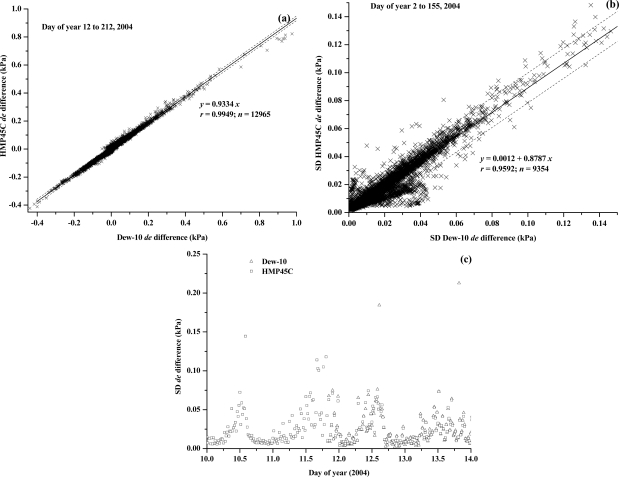
(a) and (b) Regressions of the mean of the standard deviation of the water vapour pressure profile differences (*de*) measured in the field using a HMP45C combination capacitive humidity instrument and a Dew-10 dewpoint hygrometer instruments for the periods indicated; (c) the temporal variation in *de* for a four-day period for the HMP45C and Dew-10 instruments.

The field comparison of water vapour pressure profile difference measurements for the Dew-10 and the HMP45C was very good (Figure 4(a)) during the daytime and night-time. Statistically, there is very little difference between the Dew-10 dewpoint hygrometer and HMP45C water vapour pressure profile difference measurements (Figure 4(a)). Negative measurements corresponded to *LE* directed toward the surface, and dew occurrence. The standard deviation in the water vapour pressure difference, calculated as the square root of the sum of squares of the standard deviation of the upper- and lower-level water vapour pressures, showed good agreement for the Dew-10 and HMP45C instruments (Figure 4(b)) except for small standard deviations. The standard deviations for both instruments often swamped the mean determined for each 20-min interval. The greatest standard deviations occurred during the daytime with peaks in the afternoon (Figure 4(c)).

The measured *de* values ranged between −0.45 to close to 1.0 kPa (Figure 4a). Measured |*de*| approaching 0.01 kPa were infrequent, occurring early in the morning or late in the afternoon (data not shown). Presumably, for aerodynamically rougher surfaces such as tree canopies, the magnitude of *de* and *dT* would decrease and therefore the separation distance between the upper and lower arms may need to be increased. This increase may require a greater fetch, limiting use of the BREB method if this is not possible.

**Figure 5.**
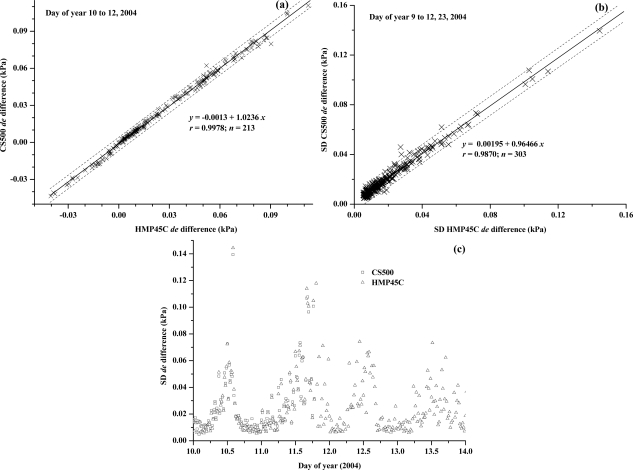
(a) and (b) Regressions of the mean of the standard deviation of the water vapour pressure profile differences (*de*) measured in the field using CS500 and HMP45C instruments for the periods indicated; (c) the temporal variation in *de* for a four-day period for CS500 and HMP45C instruments.

Measurement comparisons of *de* for the HMP45C and CS500 instruments were also good (Figure 5(a)), although for a shorter period, with good agreement in the standard deviation of *de* (Figure 5(b)). As was the case for the first field experiment, the standard deviation in the water vapour pressure profile differences increased during the daytime with spikes usually in the afternoon (Figure 5(c)).

**Figure 6.**
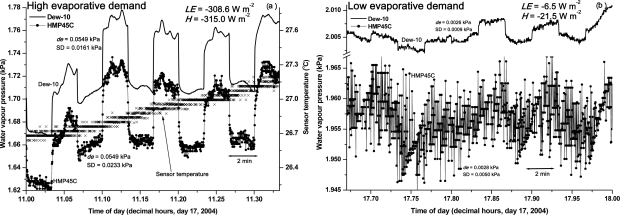
One-second measurements of water vapour pressure for a 20-min period for Dew-10 and HMP45C instruments during conditions of: (a) high evaporative demand. Also shown, for the right-hand *y*-axis, is the block temperature, which illustrates the resolution problem; (b) low evaporative demand.

In a separate field experiment, the 1-s water vapour pressures for the cooled dewpoint hygrometer and combination instrument were stored while the systems functioned normally switching air intake from the upper to the lower intake positions. In Figure 6a, the variation in Dew-10 and HMP45C water vapour pressures show an almost square-wave response, the peak-to-peak magnitude corresponding to *de*, with the upper part of the square wave corresponding to *e*_1_ for the lower position and the lower part to *e*_2_ (Equation 4).

Typically, the 20-min standard deviation of the profile differences in water vapour pressure is nearly 50 % greater for the HMP45C than for the Dew-10 (0.0233 kPa *vs* 0.0161 kPa respectively) for high evaporative demand conditions (Figure 6a) and more than five times greater (0.0050 kPa *vs* 0.0009 kPa respectively) for the low evaporative demand conditions (Figure 6b).

**Figure 7.**
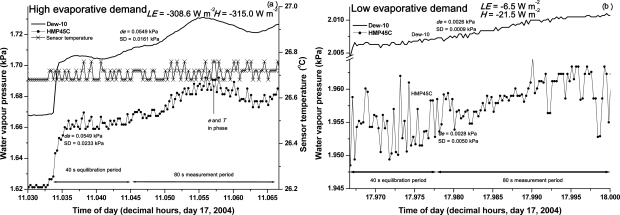
One-second variation in water vapour pressure for Dew-10 and HMP45C instruments (a) for a 40-s equilibration period followed by an 80-s averaging period for high evaporative demand conditions. The variation in block temperature is also shown; (b) for low evaporative demand conditions.

Both the Dew-10 and HMP45C instruments responded within 10 s to an abrupt change in the BREB system water vapour pressure (Figure 7(a)). The dewpoint hygrometer responded slightly more quickly (within 5 s) than the HMP45C that responded within 10 s (Figure 7(a),(b)). However this advantage and the advantage of decreased variability of the dewpoint hygrometer measurements do not disqualify the use of the HMP45C for this application since measurements are averaged over the last 80 s of a 2-min period following which the difference in average water vapour pressure between the two levels, corresponding to the latest and the previous 2-min average, is calculated.

Generally, the greatest change in water vapour pressure is at the beginning of the 2-min period and not during the 80-s averaging period. In some cases, in particular for low evaporative demand conditions, HMP45C measurements showed a large variation in water vapour pressure during the 40-s equilibration period but the average de values for the two instruments were in agreement (Figure 7b).

A report by the World Meteorological Organisation [37] gives the time constant of Vaisala Intercap/Humicap humidity sensors such as that used in the HMP45C/CS500/HMP50 instruments as 0.5 s at 25 °C (and 10 s at −40 °C) for a ventilation speed of 5 m s^−1^. The report did not state if this time constant was with the filter removed. Presumably it was since Vaisala state a 90% time constant of 15 s with the membrane filter in place.

In the case of BREB capacitive humidity measurements, since the water vapour pressure is calculated from relative humidity and temperature from an adjacent temperature sensor, it is not only the time response of the humidity sensor that is of concern. The temperature sensor time constant was only considered as a possible limitation after completion of these measurements and not fully addressed. Apart for the temperature sensor of the CS215, which has a 63% time constant of 120 s with the filter in place, the temperature sensor time constant is not specified by the manufacturer for the instruments used. The influence of the temperature sensor time constant could be reduced by using a higher flow rate. Alternatively, the temperature sensor could be replaced by a thermocouple that responds more quickly to temperature changes. However, whatever instrument is used, no adjacent temperature sensor apart from an infrared thermometer could measure the temperature of the humidity sensor although the relative humidity and temperature sensors attached to the same chip, as is the case for the CS215, should almost be at the same temperature. It is the temperature of the humidity sensor that is required together with the measured relative humidity to calculate the water vapour pressure. The possible impact of any time constant problem due to the relative humidity or temperature sensors could be reduced by increasing the 40-s stabilization period before measurements are made in the chamber. Increasing the stabilization time from 40 to 60 s should not materially affect measurement accuracy. The disadvantage of this increase is that evaporation measurements are only collected 50 % of the time for each BREB measurement height at a time.

The variation in HMP45C-measured temperature in the chamber is shown for a 20- and 2-min period (Figures 6a and 7a, respectively) for high evaporative demand conditions. The variation in the sensor temperature measurements is within 0.05 °C (Figure 7a) for the 2-min period and, for the measured relative humidity of 46.7 %, this variation would result in a variation of 0.005 kPa in water vapour pressure even if there was no variation in measured relative humidity. A variation of 0.005 kPa in water vapour pressure is reasonably consistent with the variation in water vapour pressure shown in Figure 6b, for low evaporative demand conditions. The variation in measured temperature and water vapour pressure are in-phase (Figure 7a), largely through the influence that temperature has on the calculated water vapour pressure. The in-phase variation is due to the fact that the water vapour pressure of the airstream, *e_airstream_*, is estimated using:

(20) $$e_{\mathrm{airstream}}=RH_{\mathrm{sensor}}\times e_{s}(T_{\mathrm{sensor}})/100$$

where *RH_sensor_* is the sensor-measured relative humidity and *e_s_*(*T_sensor_*) the saturated water vapour corresponding to the sensor temperature *T_sensor_*. Therefore, assuming that *RH_sensor_* is unchanged, an increase in *T_sensor_* results in a non-linear increase in *e_s_*(*T_sensor_*) causing an increase in *e_airstream_*. Similarly, a decrease in *T_sensor_* results in a decrease in *e_s_*(*T_sensor_*) causing a decrease in *e_airstream_*.

The step-like variation in temperature (Figures 6a, 7a) is due to the 21X datalogger voltage resolution limitation for the 5 V voltage range. The resolution magnitude for this range is 0.33 mV and this translates into a temperature resolution limit of 0.033 °C for the HMP45C temperature sensor. For other dataloggers from the same manufacturer (CR10X, CR800, CR1000), a 2.5 V range may be used but the resolution is still 0.33 mV. The limitation of the voltage resolution on the temperature measurements affecting the water vapour pressure estimates could be avoided by using an alternative temperature sensor that does not suffer from the same problem. The replacement sensor should also be small since it would need to be mounted very close to the relative humidity sensor. A thermocouple satisfies all of these requirements but this suggested replacement was only pursued at the very end of this study. The datalogger resolution magnitude for thermocouple measurements is 0.006 °C compared to 0.033 °C for the HMP45C temperature sensor. Some preliminary tests showed that a thermocouple exhibited a greater variation in temperature than the HMP45C temperature sensor, mainly due to the small size of the thermocouple used. This greater temperature variation caused a greater variation in the estimated water vapour pressure than if the HMP45C temperature sensor were used.

The water vapour pressure measurements of the CS500 are more variable than those obtained using the Dew-10 dewpoint hygrometer or the HMP45C (data not shown). The reason for this could be that the CS500 temperature sensor is less accurate than that used for the HMP45C (Figure 1). For example, at 20 °C, the CS500 temperature sensor has more than double the error magnitude of the HMP45C temperature sensor. The increased error in temperature measurement directly affects the error in water vapour pressure measurement, as shown previously.

With the procedures specified and based on the data collected, it would appear that the HMP45C may be used as a substitute for the dewpoint hygrometer. Field measurements of water vapour pressure profile differences, with a resolution magnitude of at least 0.01 kPa, were possible using a capacitive humidity instrument (Figures 4(a), 5(a)).

An advantage of the HMP45C is that it does have a switch control option to turn the instrument on, using datalogger control, just before measurements and then immediately off. The CS500 does not have a built-in switch control option and this results in an increased current drain if the instrument is continuously powered. This disadvantage for the CS500 is obviated when using a CR10X, CR23X, CR800 or CR1000 that can provide for switched 12 V control. However, the HMP50 (almost equivalent to the CS500) is cheaper than the HMP45C and the Dew-10 (Table 1).

### 4.3. Flux Comparisons

**Figure 8.**
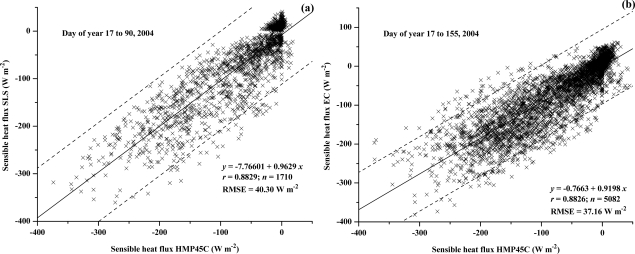
Regression of 20-min field-measured BREB sensible heat flux obtained using (a) a CS500 and (b) HMP45C instrument compared to a Dew-10 instrument for the period 17 January to 3 June 2004.

A relatively long-term measurement comparison between *H* obtained using capacitive humidity instruments and a Dew-10 dewpoint hygrometer is shown (Figure 8). As expected, the comparisons using the CS500 were more variable presumably due to increased error in the temperature measurement (Figure 8(a)). The comparison is good for the HMP45C with little statistical difference between the estimates (Figure 8(b)). The HMP45C is cheaper, more reliable, requires much less attention than the Dew-10 dewpoint hygrometer and has a reduced instrument power requirement.

**Figure 9.**
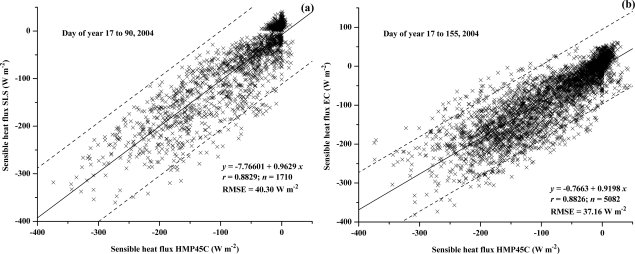
Regression of 20-min field-measured BREB sensible heat flux obtained using a HMP45C (a) against the SLS-measured *H*; (b) against the EC-measured *H*.

The comparison between *H* measured using the BREB method using the HMP45C and the SLS (Figure 9(a)) and EC method (Figure 9(b)) are reasonable, with a RMSE of about 38 W m^−2^ and a slope value of less than 1. Of note is that the BREB estimate of *H* requires *R_net_* and *S* measurements (Equation 7) whereas these are not required for the EC or SLS measurements of *H*. Besides measurement errors, measurement differences for the different methods are due to the differing theoretical bases for the methods, the spatial separation of the measurement systems, differing measurement heights, differing footprints and that the SLS measurements represent a path-weighted estimate [33,34] as well as the influence of condensing events on the different measurement methods, including the influence of dew on the net irradiance.

**Figure 10.**
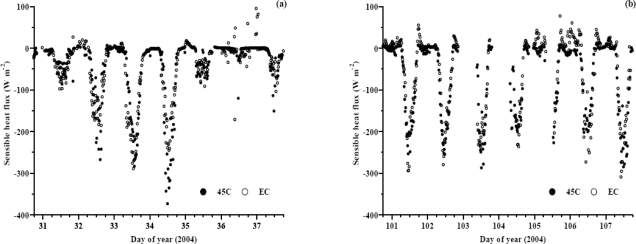
Temporal variation of 20-min field-measured EC and BREB sensible heat flux obtained using a HMP45C (a) for day of year 31 to 38 (2004); (b) for day of year 101 to 108.

Temporal plots of *H* for the EC and BREB methods, for days ranging from wet and overcast to cloudless conditions, also show reasonable correspondence. For some days (101 and 102 and 104 to 106, Figure 10b), the BREB method underestimated *H* compared to EC whereas on other days (32 and 34, Figure 10a; 103 of Figure 10b), the BREB method overestimated *H*. During the night-time, usually when dew occurs, fluctuations occur in the data particularly for the BREB method (day of year 31 to 35, Figure 10(a)) and occasionally for the EC method (day of year, Figure 10(a)).

For BREB calculations of *H* and *LE*, it is recommended that for water vapour pressure measurements, either the HMP45C be used, or a combination polymer capacitive instrument with similar or better accuracy for both *RH* and *T*. Alternatively, the CS500 or HMP50 could be used but with a more accurate temperature sensor.

## 5. Summary and Conclusions

Based on the laboratory and field experimental measurements, a combination polymer capacitive humidity instrument such as the HMP45C with a relative humidity magnitude accuracy of 2% or better and a temperature accuracy of at most 0.3 °C, perhaps 0.4 °C, is a reasonable substitute for the cooled mirror dewpoint hygrometer of a BREB system for measuring water vapour pressure profile differences. A less accurate combination capacitive humidity instrument, the CS500 or HMP50, with an accuracy of 3% and 0.4 °C for *RH* and *T* respectively, yielded less accurate water vapour pressure differences with more measurement noise and an increased RMSE of 34.89 W m^−2^ in the sensible heat flux comparisons obtained using a dewpoint hygrometer compared to a RMSE of 8.06 W m^−2^ using the HMP45C. Modifications to the combination capacitive humidity instrument included removal of the instrument membrane filter and providing a more thermally stable environment for the combination instrument chamber and adjacent connecting hoses. This substitution makes the BREB system much cheaper with little servicing required by the capacitive humidity instrument compared to the dewpoint hygrometer used. The combination capacitive humidity instruments show more water vapour pressure noise than the dewpoint hygrometer but the 20-min average of the profile water vapour pressure differences compare well with the corresponding dewpoint hygrometer measurements. Further modifications could include increasing the equilibration time from 40 s to 60 s and using a thermocouple to measure the temperature adjacent the capacitive humidity sensor or using a single-chip relative humidity and temperature capacitive instrument placed in a chamber. Sensible heat flux obtained using a HMP45C instrument compared to either surface-layer scintillometer or eddy covariance estimates yielded a RMSE of about 38 W m^−2^ and a slope less than 1.

## Figures and Tables

**Table 1.** Information and specifications of humidity instruments used or evaluated.

**Instrument**	**Description**	**Manufacturer**	**Specification**	**Approximate cost (USD, April 2010)**
LI610	Reference dewpoint generator	LI610, Li-Cor Inc., Lincoln, NE, USA	Accuracy of ±0.2 °C dewpoint	7,600
Dew-10	Dewpoint hygrometer	General Eastern Instruments, Wilmington, MA, USA	Accuracy of ±0.5 °C dewpoint, resolution of ±0.003 °C (equivalent to ±0.01 kPa)	1,610
HMP45C	RH/T capacitive	Campbell Scientific Inc., Logan, UT, USA	Error magnitude of 2% *RH* for *RH* between 10 and 90%; temperature error magnitude varies with temperature—minimum 0.2 °C at 20 °C and maximum of 0.3 °C at 0 and 40 °C	635
HMP35C (replaced by HMP45C)	RH/T capacitive	Campbell	Error magnitude of 2% *RH* for *RH* between 10 and 90%; temperature error magnitude varies with temperature–minimum of 0.4°C at 0 °C	
CS500 (equivalent to HMP50)	RH/T capacitive	Campbell	Error magnitude of 3% *RH* for *RH* between 10 and 90%; temperature error magnitude varies with temperature—minimum of 0.3 °C at 0 °C and maximum of 0.62 °C at 40 °C	425
Instruments not used				
CS215	RH/T capacitive	Campbell (based on model SHT75 from Sensirion AG, Zurich, Switzerland)	Similar to HMP35C	325
Hygroclip SC04/SC05	RH/T capacitive	Rotronic, Bassersdorf, Switzerland	Error magnitude of 1.5% *RH* for *RH* between 10 and 90%; temperature error magnitude of 0.3 °C	350
HMP155A	RH/T capacitive	Vaisala, Campbell	*δRH* = ±(1+0.008*RH)*% and *δT =* ±(0.226 − 0.0028*T*) °C for *T* < 20 °C or *δT =* ±(0.055+0.0057*T*) °C for *T*≥20°C	625
